# Validation of a Modified Child-Turcotte-Pugh Classification System Utilizing Insulin-Like Growth Factor-1 for Patients with Hepatocellular Carcinoma in an HBV Endemic Area

**DOI:** 10.1371/journal.pone.0170394

**Published:** 2017-01-20

**Authors:** Dong Hyeon Lee, Jeong-Hoon Lee, Yong Jin Jung, Jungsoo Gim, Won Kim, Byeong Gwan Kim, Kook Lae Lee, Yuri Cho, Jeong-Ju Yoo, Minjong Lee, Young Youn Cho, Eun Ju Cho, Su Jong Yu, Yoon Jun Kim, Jung-Hwan Yoon

**Affiliations:** 1 Department of Internal Medicine, Seoul Metropolitan Government Seoul National University Boramae Medical Center, Seoul, Republic of Korea; 2 Department of Internal Medicine and Liver Research Institute, Seoul National University College of Medicine, Seoul National University Hospital, Seoul, Republic of Korea; 3 Institute of Health and Environment, Seoul National University, Seoul, Republic of Korea; 4 Department of Internal Medicine, CHA Gangnam Medical Center, CHA University, Seoul, Republic of Korea; 5 Department of Gastroenterology and Hepatology, Soonchunhyang University School of Medicine, Bucheon, Republic of Korea; 6 Department of Internal Medicine, Kangwon National University Hospital, Chuncheon, Republic of Korea; Chang Gung Memorial Hospital Kaohsiung Branch, TAIWAN

## Abstract

**Background:**

Recently, a modified insulin-like growth factor-1 (IGF)–Child-Turcotte-Pugh (CTP) classification was proposed to improve the original CTP classification. This study aimed to validate the new IGF-CTP classification system as a prognostic maker for patients with hepatocellular carcinoma (HCC) in a hepatitis B virus endemic area.

**Methods:**

We conducted a post-hoc analysis of a prospective cohort study. We used Harrell’s C-index and U-statistics to compare the prognostic performance of both IGF-CTP and CTP classifications for overall survival. We evaluated the relationship between HCC stage and the four components of the IGF-CTP classification (serum levels of IGF-1, albumin, and total bilirubin and prothrombin time [PT]) using nonparametric trend analysis.

**Results:**

We included a total of 393 patients in this study. In all, 55 patients died during the median follow-up of 59.1 months. There was a difference between IGF-CTP class and CTP class in 14% of patients. Overall, the IGF-CTP classification system had a higher prognostic value (C-index = 0.604, 95% confidence interval [CI] = 0.539–0.668) than the CTP system (C-index = 0.558, 95% CI = 0.501–0.614), but the difference was not statistically significant (*P* = .07 by U-statistics). A lower serum level of IGF-1 was related to a more advanced cancer stage (*P* < .01). The remaining components of the IGF-CTP classification were not significantly related to tumor stage (*P* = .11 for total bilirubin; *P* = .33 for albumin; and *P* = .39 for PT).

**Conclusions:**

The IGF-CTP classification was slightly better than the original CTP classification for predicting survival of patients with HCC in a chronic hepatitis B endemic area. This is most likely due to the fact that serum IGF-1 levels reflect underlying HCC status.

## Introduction

Most cases of hepatocellular carcinoma (HCC) occur in the setting of chronic hepatitis and liver cirrhosis. Because of these co-existing underlying liver diseases, the functional reserve of the liver, in addition to tumor status, is a major prognostic factor in patients with HCC. The Child-Turcotte-Pugh (CTP) classification system has been the most widely accepted prognostic model for evaluating functional hepatic reserve.[1] The CTP classification is based on three objective variables (total bilirubin, albumin, and prothrombin time [PT]) and two subjective variables (ascites and encephalopathy). CTP class has been confirmed as a valuable surrogate marker for survival in many independent cohorts of patients with liver disease.[2, 3] However, the inclusion of ascites and encephalopathy allows for inter-observer variation, which has led clinicians to question the reproducibility of the CTP score. Thus, the need for a more accurate prognostic model has prompted the search for more objective markers of liver function in HCC.[4, 5]

Insulin-like growth factor (IGF)-1 is a hormone that is mainly synthesized by the liver. IGF-1 plays a critical role in childhood growth and anabolic metabolism.[6] Baseline levels of plasma IGF-1 correlate with the severity of liver disease, even in patients with HCC.[7–9] Therefore, Kaseb *et al* constructed a modified CTP classification system (IGF-CTP) to predict survival in patients with HCC by replacing the two subjective parameters in the traditional CTP system with IGF-1 level (S1 Table).[10] They observed that the IGF-CTP classification provided better risk stratification than the original CTP classification in both American and international validation cohorts.[10, 11] However, the value of the IGF-CTP classification for prognostic stratification has not been evaluated in chronic hepatitis B (CHB) endemic areas, such the Asia-Pacific region.

The aims of the current study were 1) to validate the IGF-CTP classification system in patients in a CHB endemic area by comparing the accuracy of the IGF-CTP and the CTP classifications for assessing overall survival (OS) after the first treatment for HCC and 2) to clarify the causes of differences between the predictive values of the classification systems.

## Methods

### Study population

We included patients who were enrolled in an ongoing prospective cohort study to identify biomarkers for predicting the prognosis of HCC at Seoul National University Hospital (Seoul, Republic of Korea). Briefly, between January 2006 and December 2012, 409 patients who were newly diagnosed with HCC were recruited to the prospective cohort. HCC was diagnosed on the basis of recommendations of the American Association for the Study of Liver Diseases.[12] Of the 409 original patients, 16 patients were excluded because of the concurrent presence of another primary cancer. The remaining 393 patients were included in the final analysis of this study. Patients’ epidemiologic data, clinical data, and blood samples were prospectively collected, and serum levels of IGF-1 were retrospectively determined. The study protocol conformed to the ethical guidelines of the World Medical Association Declaration of Helsinki and was approved by the Institutional Review Board of Seoul National University Hospital. All participants provided written informed consent prior to participation in the study.

### Baseline serum IGF-1 levels

Peripheral venous blood samples (5 mL) were collected from all patients before initial treatment for HCC. All sera were aliquoted and snap-frozen at -80°C until analysis. Serum IGF-1 levels were measured using an immunoradiometric assay according to the manufacturer’s instructions (Immunotech; Marseille, France).

### IGF-CTP classification

The IGF-CTP score comprises four laboratory values: total bilirubin, albumin, PT, and IGF-1.[10] Cut-off points for total bilirubin, albumin, and PT are identical with those in the original CTP classification. Cut-off points for IGF-1, which were previously derived from recursive partitioning methods and survival analyses, are 26 and 50 ng/mL. Serum levels of IGF-1 were scored as 1 point (> 50 ng/mL), 2 points (26 to 50 ng/mL), or 3 points (< 26 ng/mL). On the basis of the sum of all four component scores, patients were classified as having class A (4–5 points), B (6–7 points), or C (≥ 8 points) liver disease (S1 Table).

### Study outcomes and definitions

The primary endpoint of this study was to compare the prognostic performance of both classification systems for OS. OS was defined as the time from the date of diagnosis of HCC to the date of death from any cause. The secondary endpoints included OS prediction according to combination of both CTP and IGF-CTP classifications and the association of each parameter in the IGF-CTP classification with tumor stage. The cut-off date for data collection was September 30, 2015, and there were no missing values for these analyses.

### Statistical analysis

Statistical analyses were conducted with SPSS 22.0 (IBM; Chicago, IL, USA), the R statistical programming environment, Version 3.2.0 (R Foundation for Statistical Computing, Vienna, Austria), and Stata 13.1 (Stata Corp., College Station, TX, USA). A two-sided *P*-value of 0.05 or less was considered statistically significant. Pearson’s χ^2^ test was used to analyze differences among groups of categorical data. Survival curves were estimated using the log-rank test. Associations between insulin-like growth factor 1 and OS, and between both scoring systems and OS were assessed using univariate and multivariate Cox proportional hazard models. Harrell’s C-index was calculated to compare the prognostic power of each classification system in our cohort. A nonparametric trend analysis was used to evaluate possible trends in the scores of each component in the IGF-CTP classification (i.e., total bilirubin, albumin, and IGF-1, and PT) related to underlying tumor status.[13] This was done by calculating the average rank of each score for parameters in both classifications and correlating the results with American Joint Committee on Cancer (AJCC) stages. The *P*-values used in the nonparametric trend analysis are based on z-statistics.

## Results

### Patient characteristics

We included a total of 393 patients who were newly diagnosed with HCC in the final analysis. The mean age of the patients at the time of diagnosis was 56.8 ± 9.5 years and most of the patients (77.9%) were male. CHB was the most common cause of underlying liver disease (78.9%). According to the CTP score, 334 (85.0%), 57 (14.5%), and 2 (0.5%) patients were classified as having class A, B, and C disease, respectively (Table 1).

**Table 1:** Baseline characteristics.

Patients characteristic	Parameter	n (%)	Patients characteristic	Parameter	n (%)
Age, y	≤60	245 (62.3)	Cirrhosis	No	201 (51.1)
	>60	148 (37.7)		Yes	192 (48.9)
Sex	Male	306 (77.9)	CTP class	A	334 (85.0)
	Female	87 (22.1)		B	57 (14.5)
Viral hepatitis	HBV with/without HCV	310 (78.9)		C	2 (0.5)
	HCV without HBV	48 (12.2)	MELD score	<15	376 (95.7)
	None	35 (8.9)		≥15	17 (4.3)
Serum α-FP, ng/mL	<400	284 (72.3)	AJCC stage	I	219 (55.7)
	≥400	109 (27.7)		II	94 (23.9)
Tumor number	Uninodular	270 (68.7)		III A/B/C	47 (12.0)
	Multinodular	123 (31.3)		IV A/B	33 (8.4)
Tumor size, proportion of liver	≤50%	380 (96.7)	BCLC stage	0	82 (20.9)
	>50%	13 (3.3)		A	158 (40.2)
Main vessel invasion	No	384 (97.7)		B	37 (9.4)
	Yes	9 (2.3)		C	114 (29.0)
Lymph node spread	No	371 (94.4)		D	2 (0.5)
	Yes	22 (5.6)	Treatment history	Local therapy only	347 (88.5)
Extrahepatic metastasis	No	386 (98.2)		Surgery ± local therapy	43 (11.0)
	Yes	7 (1.8)		Systemic therapy ± local therapy	2 (0.5)
ALT, U/L	≤40	201 (51.1)		Best supportive care	0 (0.0)
	>40	192 (48.9)		Missing	1
AST, U/L	≤40	149 (40.1)			
	>40	223 (59.9)			

Abbreviations: α-FP, α-fetoprotein; AJCC, American Joint Committee on Cancer; ALT, alanine transaminase; AST, aspartate transaminase; BCLC, Barcelona Clinic Liver Cancer; CTP, Child-Turcotte-Pugh; HBV, hepatitis B virus; HCV, hepatitis C virus; MELD, Model for End-stage Liver Disease; n, number.

### Comparison of OS and prognostic accuracy of CTP and IGF-CTP classifications

**Fig 1:**
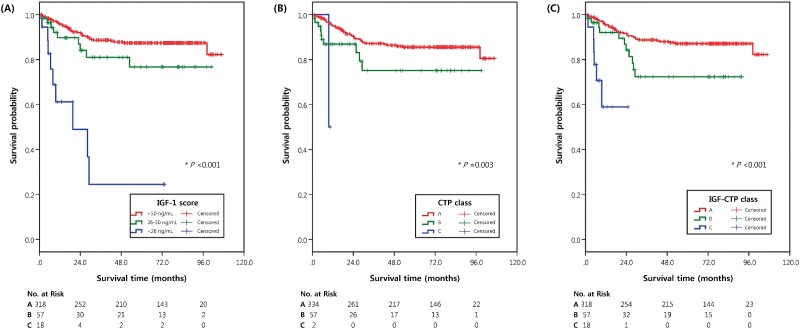
Kaplan-Meier survival curves of patients classified by serum levels of IGF-1 (A), CTP class (B), and IGF-CTP class (C). Tables below each graph show the numbers of patients at risk at various time points.

During the follow-up period (median = 59.1 months, interquartile range = 19.8–80.8 months), 55 (14.0%) patients died; the median OS was not reached. The 5-year survival rate was 84.1%. Patients with low IGF-1 levels (< 26 ng/mL) had a significantly worse prognosis than patients with intermediate (26–50 ng/mL; hazard ratio [HR] = 4.96, 95% confidence interval [CI] = 1.94–12.70, *P* < .01) and high (> 50 ng/mL; HR = 9.76, 95% CI = 4.64–20.56, *P* < .01) IGF-1 levels. Patients with intermediate IGF-1 levels also had a worse outcome than those with high IGF-1 levels. However, the difference was not significant (HR = 1.88, 95% CI = 0.91–3.90, *P* = .09) (Table 2A and Fig 1A).

**Table 2:** Cox model results for overall survival of the cohort based on insulin-like growth factor 1 (A), and both classifications (B).

**A**					
Variable	Level	n (%)	Death event	HR (95% CI)	*P*^a^
All patients		393 (100.0)			
IGF-1 level, ng/mL	1 (>50)	318 (80.9)	37	1.00 (reference)	
	2 (26–50)	57 (14.5)	9	1.88 (0.91–3.90)	0.090
	3 (<26)	18 (4.6)	9	9.76 (4.64–20.56)	<0.001
	2 (26–50)			1.00 (reference)	
	3 (<26)			4.96 (1.94–12.70)	0.001
**B**					
Classification	Class	n (%)	Death event	HR (95% CI)	*P*^a^
CTP class	A	334 (85.0)	44	1.00 (reference)	
	B	57 (14.5)	10	2.03 (1.02–4.06)	.04
	C	2 (0.5)	1	11.2 (1.48–83.90)	.02
	B			1.00 (reference)	
	C			3.45 (0.42–28.12)	.25
IGF-CTP class	A	318 (80.9)	38	1.00 (reference)	
	B	57 (14.5)	11	2.27 (1.15–4.45)	.02
	C	18 (4.6)	6	11.3 (4.44–28.60)	<.01
	B			1.00 (reference)	
	C			4.99 (1.53–16.31)	<.01

Abbreviations: CI, confidence interval; CTP, Child-Turcotte-Pugh; HR, hazard ratio; IGF, insulin-like growth factor-1; IGF-1, insulin-like growth factor-1; n, number.

^a^The univariate Cox model were used to calculate the *P* values, and the *P* values were two-sided.

OS was significantly associated with different hepatic reserve classes according to both classification systems (Table 2B and Fig 1B and 1C). The observed 5-year survival rates after curative treatment for CTP class A, B, and C disease were 85.5%, 75.1%, and 50.0%, respectively; the 5-year survival rates for IGF-CTP class A, B, and C disease were 87.1%, 72.3%, and 58.9%, respectively. The C-index of the IGF-CTP classification system (0.604, 95% CI = 0539–0.668) was higher than that of the original CTP classification system (0.558, 95% CI = 0.501–0.614), although the difference was not significant (*P* = .07) (Table 3). The Barcelona Clinic Liver Cancer (BCLC) staging system was modified by replacing CTP class with IGF-CTP class, and the C-index of the modified BCLC stage (0.732, 95% CI = 0.586–0.787) was also higher than that of original BCLC stage (0.698, 95% CI = 0.554–0.842). However, this difference was also not significant (*P* = .21).

**Table 3:** Ranking of classification by C-index.

Classification	C-index	(95% CI)	*P*^a^
IGF-CTP score	0.604	(0.539–0.668)	
CTP score	0.558	(0.501–0.614)	.07

Abbreviations: CI, confidence interval; C-index, concordance index; CTP, Child-Turcotte-Pugh; IGF, insulin-like growth factor-1.

^a^U-statistics were used to calculate *P* value, and *P* value was two-sided.

### Reassignment of patients from CTP class to IGF-CTP class

We analyzed the differences in patient distribution of the two classification systems (S2 Table). For 86.0% (338/393) of patients, there was no difference between CTP class and IGF-CTP class. Most (77.9%; 306/393) patients were classified as class A by both CTP and IGF-CTP classification systems.

**Fig 2:**
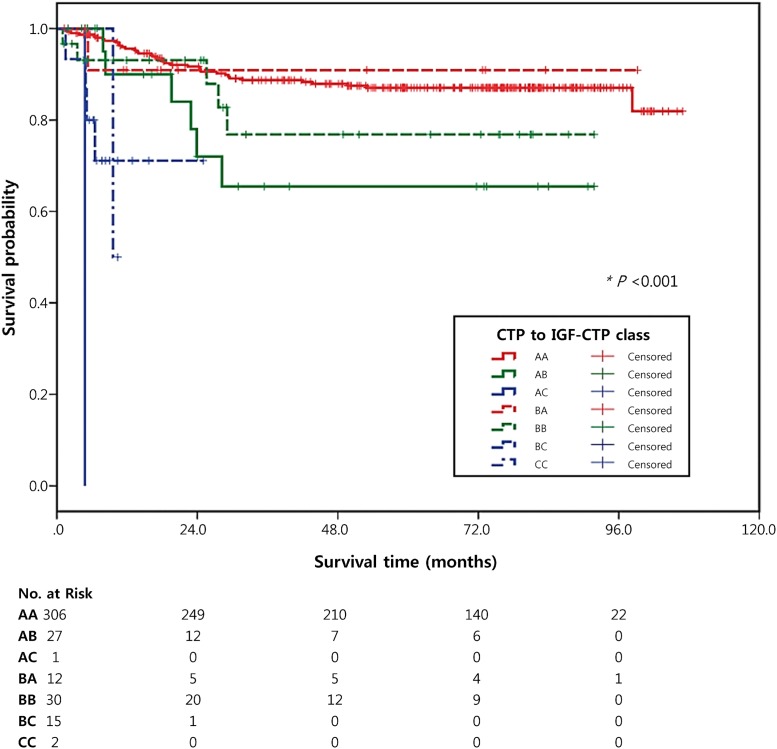
Kaplan-Meier survival curves of patients according to CTP class and IGF-CTP class. Tables below each graph show the numbers of patients at risk at various time points.

According to our results and the Kaplan-Meier curves (Table 4 and Fig 2), the IGF-CTP classification was better able to discriminate differences in subgroups than the CTP classification. CTP class A patients who were reclassified as IGF-CTP class B (AB group) or C (AC group) had a worse prognosis than patients who were reclassified as IGF-CTP class A (AA group) (HR = 2.85, 95% CI = 1.20–6.78, *P* = .02 for AB group; HR = 82.25, 95% CI = 10.22–710.85, *P* < .01 for AC group). A total of 306 (91.6%) patients were in the AA group; this group had a 5-year survival rate of 87.0%. The AB group included 27 (8.1%) patients; this group had a lower 5-year survival rate (65.5%) than the AA group. CTP class B patients who were reclassified as IGF-CTP class C (BC group) also had a worse prognosis than patients classified as class B by both the CTP and the IGF-CTP classification systems (BB group) (HR = 4.70, 95% CI = 1.10–20.16, *P* = .04).

**Table 4:** Overall survival of the cohort by whether the CTP class was reclassified IGF-CTP class.

Variable	n	Death events	HR 95% CI	*P*^a^
CTP class A / IGF-CTP class A (AA)	306	37	1.00 (reference)	
CTP class A / IGF-CTP class B (AB)	27	6	2.85 (1.20–6.78)	.02
CTP class A / IGF-CTP class C (AC)	1	1	85.25 (10.22–710.85)	<.01
CTP class B / IGF-CTP class A (BA)	12	1	0.94 (0.13–6.86)	.95
CTP class B / IGF-CTP class B (BB)	30	5	1.82 (0.72–4.65)	.21
CTP class B / IGF-CTP class C (BC)	15	4	9.06 (3.05–26.93)	<.01
CTP class C / IGF-CTP class C (CC)	2	1	14.45 (1.89–110.32)	.01
CTP class B / IGF-CTP class B (BB)			1.00 (reference)	
CTP class A / IGF-CTP class B (AB)			1.58 (0.48–5.20)	.45
CTP class A / IGF-CTP class C (AC)			52.19 (4.48–607.55)	<.01
CTP class B / IGF-CTP class A (BA)			0.58 (0.07–4.97)	.62
CTP class B / IGF-CTP class C (BC)			4.70 (1.10–20.16)	.04
CTP class C / IGF-CTP class C (CC)			6.28 (0.66–59.41)	.11
CTP class C / IGF-CTP class C (CC)			1.00 (reference)	
CTP class A / IGF-CTP class B (AB)			0.28 (0.03–2.55)	.26
CTP class A / IGF-CTP class C (AC)			21.91 (0.75–636.76)	.07
CTP class B / IGF-CTP class A (BA)			0.10 (0.01–1.76)	.12
CTP class B / IGF-CTP class C (BC)			0.80 (0.09–7.30)	.84

Abbreviations: CI, confidence interval; CTP, Child-Turcotte-Pugh; HR, hazard ratio; IGF, insulin-like growth factor-1; n, number.

^a^The univariate Cox models were used to calculate the two-sided *P* values.

### Association of each component of the IGF-CTP classification with tumor stage

Although the predictive value of the IGF-CTP classification system was not significantly better than the original CTP system, the C-index of the IGF-CTP classification was higher than that of the CTP classification in our study. To clarify the mechanism of improvement of the predictive value, the associations between AJCC stage and each parameter included in the IGF-CTP classification were analyzed (Table 5). According to a nonparametric trend analysis, a lower serum IGF-1 level was associated with more advanced HCC (*P* < .01). Total bilirubin (*P* = .11), albumin (*P* = .33), and PT (*P* = .39) were not related to AJCC stage.

**Table 5:** Results from nonparametric trend analysis evaluating possible trends between AJCC stage and each parameter of IGF-CTP classification.

Components	Score	Stage I	Stage II	Stage III	Stage IV	*P*^a^
		(n = 219)	(n = 94)	(n = 47)	(n = 33)	
		n (%)	n (%)	n (%)	n (%)	
Total bilirubin	1	201 (91.8)	81 (86.2)	40 (85.1)	30 (90.9)	.11
	2	12 (5.5)	7 (7.4)	3 (6.4)	2 (6.1)	
	3	6 (2.7)	6 (6.4)	4 (8.5)	1 (3.0)	
Albumin	1	158 (72.1)	66 (70.2)	28 (59.6)	19 (57.6)	.33
	2	46 (21.0)	24 (25.5)	19 (40.4)	12 (36.4)	
	3	15 (6.8)	4 (4.3)	0 (0.0)	2 (6.1)	
PT INR	1	217 (99.1)	91 (96.8)	47 (100.0)	32 (97.0)	.39
	2	2 (0.9)	3 (3.2)	0 (0.0)	1 (3.0)	
	3	0 (0.0)	0 (0.0)	0 (0.0)	0 (0.0)	
IGF-1	1	189 (86.3)	76 (80.9)	32 (68.1)	21 (63.6)	<.01
	2	24 (11.0)	14 (14.9)	10 (21.3)	9 (27.3)	
	3	6 (2.7)	4 (4.3)	5 (10.6)	3 (9.1)	

Abbreviations: AJCC, American Joint Committee on Cancer; CTP, Child-Turcotte-Pugh; IGF, insulin-like growth factor-1; IGF-1, insulin-like growth factor-1; INR, international normalized ratio; n, number; PT, prothrombin time.

^a^Nonparametric trend analysis was used to calculate *P* value, and *P* value was two-sided.

## Discussion

We validated the predictive value of the IGF-CTP classification system for assessing survival in HCC patients from a CHB endemic area. The C-index of the IGF-CTP classification was higher than that of the original CTP classification, but this difference was not significant. The proportion of patients who had a difference in risk stratification between the two classifications was not enough (14.0%) to establish statistical significance. Lower serum IGF-1 levels were significantly associated with more advanced HCC. These findings suggest that the IGF-CTP classification system has a tendency to predict survival more accurately than the CTP system by reflecting underlying HCC status.

Functional hepatic reserve can affect survival of HCC patients, so several HCC staging systems include CTP class when assessing disease stage.[14–17] However, the CTP class itself is also used to guide initial or subsequent treatment choices by predicting the risk of hepatic failure and death after treatment.[1] CTP class is partially based on subjective parameters, which are difficult to grade objectively and can be easily influenced by medications.[18–20] Therefore, objective classifications have been suggested, including the model for end-stage liver disease score, which has replaced CTP class in decision-making schemes for allocation of organs for patients undergoing liver transplantation.[4, 21] However, until the development of the IGF-CTP classification, no system has been established for patients with HCC.[10] The IGF-CTP classification has been validated in two independent cohorts from regions where dominant risk factors for HCC included hepatitis C virus (HCV). However, this classification has not been evaluated in cohorts of patients infected with hepatitis B virus (HBV). Since clinical outcomes vary considerably depending on the etiology of HCC[22–24] and, globally, HBV is the most common cause of HCC,[25] it is essential to validate the IGF-CTP classification in CHB endemic areas prior to the widespread application of the new classification system.

Interestingly, recent studies suggested that higher plasma IGF-1 levels were related to various malignancies, including prostate, breast, colon, and lung cancers.[26–29] However, plasma levels of IGF-1 are reduced in patients with chronic liver disease because circulating IGF-1 is synthesized in the liver.[30–33] Furthermore, other studies suggested that lower plasma levels of IGF-1 were strongly associated with more advanced HCC parameters.[34–36] Since HCC cells suppress normal hepatic function by replacing normal hepatocytes, advanced HCC might reduce IGF-1 synthesis to a greater extent than early HCC. In fact, our study showed that lower levels of serum IGF-1 were significantly related to more advanced AJCC stages. Three of the parameters included in the CTP classification (total bilirubin, albumin, PT) were not related to AJCC stage. Furthermore, patients with low IGF-1 levels (< 26 ng/mL) had a significantly worse prognosis than patients with intermediate (26–50 ng/mL) or high (> 50 ng/mL) IGF-1 levels, independent of CTP class or MELD score (S3 Table). A previous study also revealed that IGF-1 is correlated with HCC burden, after adjusting for hepatic reserves.[31] Therefore, the difference in predictive values between classification systems likely originates from the inclusion of the serum level of IGF-1 instead of scores for ascites and encephalopathy.

This was the first study to evaluate the long-term predictive value of the IGF-CTP classification system for survival in HCC patients. A major strength of our study is that this is the first validation of the IGF-CTP classification system in a CHB endemic area. In our cohort, 78.9% of total patients were infected with HBV; most patients included in previous validation cohorts were infected with HCV.[10, 11] Furthermore, the median follow-up duration of our study was at least three times longer than previous validation studies (16.5 months for a United States validation cohort and 8.6 months for an Egyptian validation cohort).[10, 11] Another strength of the present study is that this is the first study to clarify the cause of differences between predictive values the classification systems. The IGF-CTP system tended to predict survival more accurately than the CTP system by reflecting underlying HCC status. Although several validation studies were performed before our study, the performance enhancement mechanism of the IGF-CTP class has not been evaluated.

Our study has some limitations that should be considered. First, the majority of patients (77.9%) had well-compensated hepatic reserve and were classified as having class A disease by both the CTP and the IGF-CTP classifications. The proportion of patients with this “AA” designation was much higher in our study than in previous validation studies.^10,11^ Since most patients (86.0%) were designated as the same risk class by both classifications, it was impossible to establish the statistical significance of the differences between the two classification systems. The negative results of our study might originate from the fact that many of the enrolled patients had minimal risk factors for a poor HCC prognosis, rather than differences in the etiology of HCC. However, the limited discriminative function of the IGF-CTP classification in patients with mild disease is a critical weak point, considering recent trends in HCC surveillance.

## Conclusion

Although its improvement was not statistically significant compared to the original CTP classification, the IGF-CTP classification system demonstrated better discriminatory function for predicting survival of patients with HCC in a CHB endemic area. The IGF-CTP classification also considerably improved the accuracy of survival prediction in previous validation studies. Therefore, using the IGF-CTP classification instead of the CTP classification in clinical practice can help physicians properly classify patients regardless of the etiology of HCC.

## Supporting Information

S1 Table The new modified CTP (IGF-CTP) classification. (DOCX)

S2 Table Patient distribution in the cohort for IGF-CTP class by CTP class. (DOCX)

S3 Table Adjusted Cox model results for overall survival of the cohort based on insulin-like growth factor 1. (DOCX)
