# Supplementary material for: Validation of a Modified Child-Turcotte-Pugh Classification System Utilizing Insulin-Like Growth Factor-1 for Patients with Hepatocellular Carcinoma in an HBV Endemic Area
Source: PLoS One. 2017 Jan 20;12(1):e0170394. doi: 10.1371/journal.pone.0170394 (PMC5249174; doi:10.1371/journal.pone.0170394)
Supplement: S1 Table — (DOCX) [file pone.0170394.s001.docx]

**S1 Table. The new modified CTP (IGF-CTP) classification**

|  | IGF-CTP score^a^ (points) | | |
| --- | --- | --- | --- |
| Parameter | 1 | 2 | 3 |
| Total bilirubin (mg/dL)^b^ | <2 | 2-3 | >3 |
| Albumin (g/dL) | >3.5 | 2.8-3.5 | <2.8 |
| PT INR | <4 | 1.7-2.3 | >2.3 |
| IGF-1 (ng/mL) | >50 | 26-50 | <26 |

Abbreviations: CTP, Child-Turcotte-Pugh; IGF, insulin-like growth factor-1; IGF-1, insulin-like growth factor-1; INR, international normalized ratio; PT, prothrombin time.

^a^IGF-CTP class: A (4-5), B (6-7), C (>7).

^b^In primary biliary cirrhosis and primary sclerosing cholangitis, the upper limit of bilirubin for 1 point is 4 mg/dL and the upper limit for 2 points is 10 mg/dL.
