# Supplementary material for: Validation of a Modified Child-Turcotte-Pugh Classification System Utilizing Insulin-Like Growth Factor-1 for Patients with Hepatocellular Carcinoma in an HBV Endemic Area
Source: PLoS One. 2017 Jan 20;12(1):e0170394. doi: 10.1371/journal.pone.0170394 (PMC5249174; doi:10.1371/journal.pone.0170394)
Supplement: S2 Table — (DOCX) [file pone.0170394.s002.docx]

**S2 Table. Patient distribution in the cohort for IGF-CTP class by CTP class**

|  | IGF-CTP class, n (%) | | | | | | | |
| --- | --- | --- | --- | --- | --- | --- | --- | --- |
| CTP class | A | (4-5) | B | (6-7) | C | (>7) | Total | |
| A (5-6) | 306 | (77.9) | 27 | (6.9) | 1 | (0.3) | 334 | (85.0) |
| B (7-9) | 12 | (3.1) | 30 | (7.6) | 15 | (3.8) | 57 | (14.5) |
| C (>10) | 0 | (0.0) | 0 | (0.0) | 2 | (0.5) | 2 | (0.5) |
| Total | 318 | (80.9) | 57 | (14.5) | 18 | (4.6) | 393 | (100.0) |

Abbreviations: CTP, Child-Turcotte-Pugh; IGF, insulin-like growth factor-1; n, number.
