# Supplementary material for: Validation of a Modified Child-Turcotte-Pugh Classification System Utilizing Insulin-Like Growth Factor-1 for Patients with Hepatocellular Carcinoma in an HBV Endemic Area
Source: PLoS One. 2017 Jan 20;12(1):e0170394. doi: 10.1371/journal.pone.0170394 (PMC5249174; doi:10.1371/journal.pone.0170394)
Supplement: S3 Table — (DOCX) [file pone.0170394.s003.docx]

**S3 Table. Adjusted Cox model results for overall survival of the cohort based on insulin-like growth factor 1**

| IGF-1 level, ng/mL | 1 (>50) | |  | 2 (26-50) | | |  | 3 (<26) | | |
| --- | --- | --- | --- | --- | --- | --- | --- | --- | --- | --- |
|  | HR | (95% CI) |  | HR | (95% CI) | *P*^a^ |  | HR | (95% CI) | *P*^a^ |
| Model 1^b^ | 1.00 | (reference) |  | 1.88 | (0.91-3.90) | 0.090 |  | 9.76 | (4.64-20.56) | <0.001 |
|  |  |  |  | 1.00 | (reference) |  |  | 4.96 | (1.94-12.70) | 0.001 |
| Model 2^b^ | 1.00 | (reference) |  | 2.03 | (0.97-4.27) | 0.060 |  | 10.32 | (4.85-21.95) | <0.001 |
|  |  |  |  | 1.00 | (reference) |  |  | 4.86 | (1.89-12.51) | 0.001 |
| Model 3^b^ | 1.00 | (reference) |  | 1.87 | (0.87-4.03) | 0.109 |  | 9.04 | (4.02-20.32) | <0.001 |
|  |  |  |  | 1.00 | (reference) |  |  | 4.63 | (1.78-12.00) | 0.002 |
| Model 4^b^ | 1.00 | (reference) |  | 2.04 | (0.96-4.34) | 0.064 |  | 10.43 | (4.35-25.01) | <0.001 |
|  |  |  |  | 1.00 | (reference) |  |  | 4.77 | (1.69-13.46) | 0.003 |

Abbreviations: CI, confidence interval; HR, hazard ratio; IGF-1, insulin-like growth factor-1

^a^The univariate and multivariable Cox model were used to calculated the two-sided P values.

^b^Model 1: Unadjusted

Model 2: Adjusted for age and sex

Model 3: Adjusted for age, sex, and CTP class

Model 4: Adjusted for age, sex, and MELD score
